# Shall Nurses Cease to Be Laywomen?—III

**Published:** 1891-09-26

**Authors:** 


### Passing Topics.

### SHALL NURSES CEASE TO BE LAY-WOMEN ?—III.

Whenever the notion is established that nurses as members of a profession are outside the control of the lay authorities of the hospital, revolution will impend. The portentous exclusiveness demanded by, and conceded to, the medical profession has not so much to recommend it that we need seek to extend the limits of its influence. The exaggerated deference paid to the members of the staff in a hospital verges closely upon the ludicrous. It is not that every want must be anticipated and every look obeyed. Thac is all well enough. It is to the obsequious attitude which, for instance, compels to silence when speech would be useful, that we take exception. Quite naturally, the nurses would desire to exercise their own functions with similar impressiveness, a result not only—as we conceive—detrimental to the patient, but costly to the institution.

In hospital life a closer incorporation of lay and medical authority would be followed by many and great advantages, and anything which helps to render the separation more complete is to be deprecated. The position of the medical staff in its relation to the management is nearly always an example of the *imperium in imperio* condemned by the most elementary rules of government. Yet, putting aside the fact that the services of the staff are given under conditions totally dissimilar from those which obtain in respect of nurses, there are safeguards in the case of the select body of hospital physicians and surgeons it would be absurd to look for in the turbid stream of nurses which flows through the hospitals year by year.

When we pass from the question of discipline and consider the effect of undiluted professionalism in respect of the patient, we are led to the belief that its introduction will eradicate qualities which can be ill spared from the sick room. Nurses, while strictly obedient to the physician, are now able to perform useful functions, of which some are beyond his care and ken. It is easy to imagine the same woman a very good nurse so far as the physician is concerned, but entirely unacceptable to the patient. From those whose object it is to wholly incorporate the nurse with the doctor, we hear much of skill and training, but scarcely enough of sympathy and compassion. Sentimental considerations would, of course, be out of place in a strictly "scientific" body, but looked at from the patients' point of view, we can imagine few greater misfortunes than for the sick to be tended by women imbued with a professional spirit and nothing more. In place of nurses, as we now know them, we must look to find a set of women scornful of exercising those softer influences we like to associate with woman's nature; amazons prepared to battle with disease, but too much engrossed with the technical side of duty and too fully actuated by the clinical spirit to set before themselves as the first and foremost object of their labours, the relief of suffering rather than to show well in the critical conversation of the nurses' room.

There is scarcely a limit to the power the nurse has to mar or make the comfort of the patient. Mere training will not render her sympathetic and forbearing, to the disagreeable no less than the pleasant. Remove the nurse beyond lay control, and the work of the hospital must cease to be primarily philanthropic. The lay element in the administration safe-guards the humanity, and we may almost say the decency of our hospital system, and it is through the nurse the laity keeps in touch with the patient. We must bear in mind that the staff of a hospital is usually composed of men foremost in general medicine or in their several specialities—men who before all things are scientific. By such ardent investigators, facts, hard, dry facts are alone desired. To them, the mere cure of an ordinary ailment about which everything is known, albeit honestly promoted, is necessarily of less moment than the study of interesting phenomena and the clearing up of a difficult problem. Committed to the guidance of mentors like these, and set free of all corrective influences, the nurses would soon imitate the keen enthusiasm of their masters without acquiring their intellectual gravity, and, while shedding the native graces which render a woman's co-operation really grateful and beneficial to the sick, would offer nothing in its place but the certificate of a spurious professionalism. Agricola.

*(To be continued.)*

